# Comparison of the efficacy of thalidomide combined with low-dose rituximab and low-dose rituximab alone in the treatment of steroid-resistant/refractory adult patients with primary immune thrombocytopenia: an open-label trial

**DOI:** 10.3389/fmed.2025.1670736

**Published:** 2026-01-09

**Authors:** Shuo Yang, Mingfeng Zhao, Genjie Wang, Qingzhu Hu, Ying Tian, Lin Yuan, Han Shen, Junli Zhang, Chengfu Ji, Hua Zhou

**Affiliations:** 1 Department of Hematology, The First People's Hospital of Shangqiu, Shangqiu, Henan, China; 2 Department of Hematology, Tianjin First Central Hospital, Tianjin, China; 3 Department of Hematology, Funing People’s Hospital, Yancheng, Jiangsu, China

**Keywords:** ITP, lymphocyte subsets, NK cell, rituximab, thalidomide

## Abstract

**Background:**

Primary immune thrombocytopenia (ITP) is an autoimmune disorder characterized by reduced platelet counts, leading to an increased risk of bleeding and substantial impairment patients’ quality of life. Although rituximab (RTX) is commonly employed as a second-line treatment for patients with steroid-refractory or steroid-resistant ITP, its long-term efficacy as monotherapy remains limited.

**Objective:**

This study aimed to evaluate the efficacy and safety of low-dose RTX (LD-RTX) in combination with thalidomide (THD) in the treatment of patients with steroid-refractory or steroid-dependent ITP.

**Method:**

A total of 100 patients with ITP who failed first-line treatment at Shangqiu First People’s Hospital and Funing County People’s Hospital between January 2021 and January 2025 were enrolled in a randomized controlled trial. Participants were assigned to one of two groups: the LD-RTX group (ctrl-group) or the T + LD-RTX group (thal-group).

**Results:**

The overall response rate in the thal-group was significantly higher than in the ctrl-group (79.17% vs. 55.32%; *p* < 0.05). Among patients who achieved a therapeutic response, baseline levels of CD16^+^CD56^+^CD3^−^ natural killer (NK) cells were significantly lower in the complete remission (CR) group (78.81 ± 20.15 cells/μL) and partial remission (PR) group (91.82 ± 28.64 cells/μL) compared to the non-remission (NR) group (139.00 ± 42.42 cells/μL; *p* < 0.05). In the thal-group, the absolute count of CD16^+^ NK cells in responders (CR + PR) increased from 84.63 ± 24.85 cells/μL at baseline to 138.71 ± 19.20 cells/μL after 6 months. In contrast, in the ctrl-group, NK cell counts increased slightly from 92.96 ± 23.17 cells/μL to 129.73 ± 28.50 cells/μL over the same period. Patients with lower pre-treatment levels of CD16^+^CD56^+^CD3^−^ NK cells demonstrated better therapeutic responses, and a significant post-treatment increase in NK cell counts was observed in the combination group (*p* < 0.05). ROC analysis identified a threshold of 110.5 cells/μL for the absolute NK cell count (sensitivity: 81.6%; specificity: 100.0%) and 5.72% for the NK cell percentage (sensitivity: 65.8%; specificity: 100.0%) as predictive markers for achieving CR or PR. Lower baseline values were associated with more favorable outcomes.

**Conclusion:**

The combination of THD LD-RTX represents a promising therapeutic strategy for patients with steroid-refractory or steroid-dependent ITP. Furthermore, baseline NK cell levels may serve as a useful biomarker for predicting treatment response.

## 1 Introduction

Primary immune thrombocytopenia (ITP) is an autoimmune disorder characterized by a decreased platelet (PLT) count. The current first-line treatment for ITP primarily involves the use of glucocorticoids, which are cost-effective and demonstrate considerable short-term efficacy, with response rates reaching up to 60% (1). However, the long-term efficacy of glucocorticoid therapy in adults is limited, and sustained response rates range from only 20 to 30% (2, 3). Notably, ITP is a typical immune-mediated inflammatory disease (IMID) whose pathogenesis involves aberrant activation of multiple immune cells and dysregulation of the inflammatory cytokine network. In recent years, treatment strategies for IMIDs have shifted from single-target immunosuppression toward multi-target modulation. Against this backdrop, thalidomide (THD), as an important class of IMID therapeutic agents, has garnered substantial clinical experience in China. By modulating key inflammatory cytokines, such as TNF-*α* and IL-6, THD has demonstrated unique therapeutic value in various autoimmune diseases (4). Of particular concern is that although ITP patients primarily present with bleeding tendencies, the disease itself and the use of certain immunomodulatory drugs may alter the risk of thrombosis—a paradoxical phenomenon that warrants special attention in the management of IMIDs.

For patients who are refractory to or dependent on steroids, rituximab (RTX) is commonly employed as a second-line therapeutic option. RTX is a monoclonal antibody that specifically targets CD20-positive B lymphocytes, thereby reducing the production of antibodies. Although the initial response rate to RTX is approximately 50%, long-term sustained efficacy is maintained in only 30–40% of patients during follow-up assessments at 6 or 12 months post-treatment (5, 6). These limitations have driven growing interest in combination therapies that target additional immune pathways, particularly cellular immunity. THD, a glutamic acid derivative, exhibits notable immunomodulatory properties. It modulates T lymphocyte activation, influencing co-stimulatory and inhibitory signals and thereby altering immune responses. Additionally, THD regulates the expression of various cytokines, including IL-6, IL-10, IL-2, IL-12, IL-1β, and TNF-*α* (7). In the context of ITP, emerging evidence suggests that THD exerts therapeutic effects not only through modulation of Treg and Breg cells but also by influencing the function of mesenchymal stem cells (MSCs) (8, 9).

According to the *Chinese Guidelines for the Diagnosis and Treatment of Primary Immune Thrombocytopenia in Adults* (10), RTX remains a key agent for second-line ITP treatment; however, its limited sustained response rate and high cost restrict its widespread clinical use. Enhancing treatment efficacy and identifying predictive markers of response remain urgent challenges in ITP management. In this context, Wu YJ et al. (11) reported that RTX combined with all-trans retinoic acid (ATRA) significantly improved the ORR in patients with ITP, offering new therapeutic insights. Consequently, RTX-based combination therapies, including those with retinoic acid, thrombopoietin receptor agonists (TPO-RAs), and glucocorticoids, have become a focus of current research (12, 13). These regimens aim to exploit the synergistic mechanisms of action between agents to reduce relapse rates and improve long-term remission outcomes. However, the selection of an appropriate combination therapy must be individualized, taking into account multiple patient-specific factors, such as bleeding risk, disease duration, treatment-related adverse effects, age, and economic considerations. Personalized treatment strategies are essential to optimize therapeutic benefit while minimizing risk. In addition, currently, low doses of RTX are commonly used in the clinical treatment of ITP. A meta-analysis by Dong et al. (14) showed that for adult ITP, the efficacy of low doses RTX (100 mg every week for 4 weeks = 400 mg) is comparable to standard dose (1 g day 1 + day 15 = 2 g) treatment, but it has fewer adverse reactions, higher safety, more convenient treatment, and lower medical costs.

Given the distinct therapeutic mechanisms of RTX and THD in the treatment of ITP, this study aims to evaluate the efficacy and safety of their combined use at low doses of RTX combined with THD in patients with steroid-resistant or steroid-refractory ITP. This combined treatment regimen may serve as a novel treatment option for patients with ITP.

## 2 Methods

### 2.1 Research object

Patients with ITP who failed first-line treatment were enrolled from Shangqiu First People’s Hospital and Funing County People’s Hospital between January 2021 and January 2025. The inclusion criteria were as follows: (1) age ≥ 18 years; (2) diagnosis consistent with the latest international consensus report on the investigati006Fn and management of primary Immune Thrombocytopenia (2); (3) documented failure of first-line therapy. Steroid-refractory ITP was defined as a lack of response to two courses of high-dose dexamethasone 40 mg/day for 4 days or prednisone at 1 mg·kg^−1^·day^−1^ for 2 weeks. Steroid-dependent ITP was defined as requiring >5 mg/day of prednisone or intermittent corticosteroid administration to maintain a PLT count ≥30 × 10^9^/L or to prevent bleeding. Other first-line regimens included steroid-based combinations with TPO-RAs or intravenous immunoglobulin. Exclusion criteria included: (1) secondary thrombocytopenia of any etiology; (2) comorbid autoimmune diseases, thyroid disorders, lymphoproliferative diseases, myelodysplastic syndromes, aplastic anemia, malignant hematologic disorders, tumor infiltration, chronic liver disease, hypersplenism, common variable immunodeficiency, infections, or vaccination-induced thrombocytopenia; (3) thrombocytopenia caused by PLT consumption, drug-induced mechanisms, alloimmune causes, pregnancy, pseudothrombocytopenia, or congenital conditions; (4) severe organ dysfunction, peripheral neuropathy, active infections, primary immunodeficiency, venous thrombosis within the previous 6 months, or current pregnancy, lactation, and planned pregnancy patients; (5) prior exposure to RTX. Eligible patients were randomly assigned to either the low-dose RTX (LD-RTX) or the THD combined with low-dose RTX (T + LD-RTX) group.

### 2.2 Grouping method and medication method

Randomization was conducted by independent statisticians using a computer-generated random number sequence. Treatment allocation was determined according to a pre-established randomization list, which was centrally maintained by the coordinating center at Shangqiu First People’s Hospital. To ensure allocation concealment, the sealed envelope method was employed: Each random number and its corresponding group assignment were enclosed in an opaque, sealed envelope labeled only with a serial number. After obtaining written informed consent, investigators opened the envelopes sequentially to assign patients to treatment groups. Eligible patients were randomized in a 1:1 ratio to one of the following two treatment arms:

LD-RTX group (ctrl-group): Patients received a fixed dose of RTX (100 mg) administered via intravenous infusion once weekly for a total of four doses.

T + LD-RTX group (thal-group): In addition to the LD-RTX regimen described above, patients received THD (100 mg) orally once daily. After 3 months, the dose was reduced to 50 mg once daily and continued for an additional 3 months, totaling 6 months of THD therapy.

This study was designed as an open-label trial, in which both patients and treating physicians were aware of group assignments. However, to minimize assessment bias, data collection and statistical analyses were conducted by personnel blinded to treatment allocation.

For patients with ineffective treatment, oral ATRA (10 mg/time, twice a day) is administered. Rescue treatment was performed in combination with danazol (200 mg/time, twice a day) for a total of 16 weeks.

### 2.3 Observation indicators and efficacy evaluation

#### 2.3.1 Clinical data collection

Clinical data were collected from inpatient and outpatient records and included gender, age, disease duration, medical history, physical examination, whole blood count, baseline platelet count, previous treatment history, blood routine, urine routine, biochemical parameters, liver and kidney function, blood smear, bone marrow smear, and other data.

#### 2.3.2 Number of platelets (PLTs)

PLT counts were recorded for both groups before the start of treatment and after 6 months of treatment. Treatment response was categorized according to the following criteria: complete response (CR): PLT count ≥100 × 10^9^/L with no clinical evidence of bleeding. Partial response (PR): PLT count ≥30 × 10^9^/L with no bleeding, or a PLT count at least twice the baseline level. Non-remission (NR): PLT count <30 × 10^9^/L or failure to achieve a two-fold increase from baseline. Overall response (OR): Defined as a PLT count ≥30 × 10^9^/L without the need for additional ITP-specific treatment and no bleeding observed on multiple blood routine tests within 6 months of enrollment (15). Bleeding severity was assessed using the specific ITP bleeding classification system (16), where grade 0 indicates no bleeding, grade 1 denotes petechiae, grade 2 represents mild blood loss, grade 3 corresponds to significant blood loss, and grade 4 reflects disabling or life-threatening bleeding.

#### 2.3.3 Changes in lymphocyte subpopulations

The absolute counts and percentages of lymphocyte subsets, including total circulating lymphocytes, CD3^+^ T cells, CD4^+^ helper T cells, CD8^+^ cytotoxic T cells, CD19^+^ B cells, and CD16^+^CD56^+^CD3^−^ natural killer (NK) cells, were measured using a Beckman Coulter FC500 flow cytometer both before the start of treatment and after 6 months of treatment (17).

### 2.4 Ethics

An open-label, randomized clinical trial was conducted involving patients diagnosed with ITP at Shangqiu First People’s Hospital and Funing County People’s Hospital between January 2021 and January 2025. The study protocol was reviewed and approved by the Ethics Committees of Shangqiu First People’s Hospital (Approval No. HS2021031) and Funing County People’s Hospital (Approval No. 2020-L-043). All participants provided written informed consent before enrollment in accordance with the principles outlined in the Declaration of Helsinki.

### 2.5 Statistical methods

Statistical analyses were performed using SPSS version 20.0 (IBM Corp., Armonk, NY), and data visualization was conducted using GraphPad Prism version 6.0 (GraphPad Software, San Diego, CA). Categorical variables were expressed as frequencies and percentages, while continuous variables were reported as either mean ± standard deviation (SD) or median with interquartile range (IQR), depending on data distribution. For between-group comparisons, *χ*^2^ test or Fisher’s exact test was used for categorical variables. Ordinal variables, such as bleeding scores, were analyzed using the Mann–Whitney U test. Treatment efficacy outcomes (CR, PR, and NR) and incidence of adverse reactions were compared using the *χ*^2^ test, and proportional differences were reported alongside their 95% confidence intervals (CIs). Lymphocyte subset data before and after treatment (thal-group or ctrl-group) were stratified by response category and tested for normality using the Shapiro–Wilk test. For normally distributed data, one-way analysis of variance (ANOVA) was applied, whereas non-normally distributed data were analyzed using the Kruskal–Wallis test. *Post hoc* pairwise comparisons for nonparametric data were performed using the Mann–Whitney U test, with multiple testing corrections applied using the Benjamini–Hochberg procedure. Paired comparisons of lymphocyte subsets before and after treatment were conducted using the paired sample t-test for normally distributed data and the Wilcoxon signed-rank test for non-normally distributed data. ROC curves were generated to assess the predictive performance of absolute counts and percentages of CD16^+^CD56^+^CD3^−^ NK cells in distinguishing responders (CR + PR) from non-responders, and to determine optimal cutoff values. All tests were two-tailed, and a *p* value <0.05 was considered statistically significant.

## 3 Results

### 3.1 Comparison of baseline characteristics of patients

Between January 2021 and January 2025, a total of 1,429 ITP patients were diagnosed, including 314 patients under 18 years old, 586 patients who were effective in first-line treatment, 124 patients with secondary ITP, 74 pregnant women, and 231 patients with other diseases and contraindications. Finally, a total of 100 patients who were diagnosed with ITP and had failed first-line therapy were enrolled and randomly assigned to either the ctrl-group (*n* = 50) or the THD combined with LD-RTX group (thal-group, *n* = 50). During the treatment period, three patients in the ctrl-group discontinued participation: two gave up treatment midway and one withdrew due to early discharge. Similarly, two patients in the thal-group withdrew: one gave up treatment midway and one withdrew due to early discharge. Ultimately, 95 patients completed the treatment protocol and were included in the final statistical analysis, comprising 47 patients in the ctrl-group and 48 in the thal-group. The baseline characteristics of the enrolled patients are summarized in Table 1. In the thal-group, 16 patients (33.33%) were male and 32 (66.67%) were female, with a median age of 44.49 years. In the ctrl-group, 20 patients (42.55%) were men and 27 (57.45%) were women, with a median age of 45.26 years. All patients received corticosteroids as first-line treatment; 13 (13.68%) received corticosteroids in combination with intravenous immunoglobulin, while 64 (67.37%) received corticosteroids in combination with TPO-RAs. A total of 36 patients (37.89%) were classified as steroid-dependent, and 59 patients (62.11%) as steroid-resistant. The median PLT count before treatment was 18.5 × 10^9^/L in the ctrl-group and 17.0 × 10^9^/L in the thal-group. No statistically significant differences were found between the two groups in terms of gender distribution, age, prior treatment regimens, steroid dependency status, baseline PLT count, or baseline bleeding scores (all *p* > 0.05). Additionally, analysis of lymphocyte subset profiles prior to treatment revealed no significant intergroup differences in total circulating lymphocyte counts, percentages of CD3^+^ T cells, CD4^+^ helper T cells, CD8^+^ cytotoxic T cells, CD19^+^ B cells, or the percentage and absolute count of CD3^−^CD16^+^CD56^+^ NK cells (all *p* > 0.05).

**Table 1:** Baseline characteristics of patients enrolled in the study.

Parameters	ctrl-group (*n* = 47)	thal-group (*n* = 48)	Test statistic value	*p*
Gender (Male/Female)	20/27	16/32	0.158^a^	0.691
Age [M(P25, P75), years]	47.0 (30.0–57.0)	44.5 (27.50–55.00)	−0.908	0.782
Treatment regimen before enrollment [*n* (%)]				
Glucocorticoids	47 (100.00)	48 (100.00)	-	-
Human immune disease globulin	5 (10.64)	8 (16.67)	0.731^a^	0.393
rhTPO	35 (74.47)	29 (60.42%)	2.133^a^	0.144
Hormonal response [*n* (%)]			0.158^a^	0.691
Steroid-dependent	20 (42.55)	16 (33.33)		
Steroid-resistant	27 (57.45)	32 (66.67)		
Platelet count [M(P25, P75), 10^9^/L]	17.00 (14.00–22.00)	18.5 (16.00–22.00)	0.471	0.211
Bleeding score [*n* (%)]			4.519	0.340
0	13 (27.66)	9 (18.75)		
1	21 (44.68)	20 (41.67)		
2	11 (23.40)	11 (22.92)		
3	2 (4.26)	7 (14.58)		
4	0 (0)	1 (2.08)		
Absolute value of lymphocyte subpopulations before treatment (cells/ul) Mean±SD				
Circulating lymphocytes	1672.59 ± 225.16	1723.56 ± 348.21	0.847	0.399
CD19^+^ B cells	335.43 ± 95.61	374.77 ± 143.83	1.656	0.101
CD3^+^ T cells	1189.94 ± 200.72	1213.58 ± 302.66	0.45^b^	0.654
CD3 + CD4^+^ T helper cells	668.22 ± 186.19	644.73 ± 197.94	0.755	0.452
CD3 + CD8^+^ cytotoxic T cells	610.38 ± 243.45	602.37 ± 243.20	−0.771	0.443
CD16^+^CD56^+^CD3^−^NK cells	110.55 ± 59.12	95.95 ± 36.45	−1.882	0.063

ctrl-group = low-dose rituximab group, thal-group = thalidomide combined with low-dose rituximab group, rhTPO = recombinant human thrombopoietin Indicate the *χ*^2^ value, ^b^ indicates *Z* value, and the rest are *t* values.

### 3.2 Comparison of the therapeutic effects of patients

At 6 months post-treatment, treatment efficacy was assessed on the basis of PLT count recovery and bleeding status. In the thal-group, 21 patients (44.68%) achieved a CR, 17 patients (37.13%) achieved a PR, and 10 patients (21.66%) exhibited no response. By contrast, in the ctrl-group, 7 patients (14.89%) achieved CR, 19 patients (40.43%) achieved PR, and 21 patients (44.68%) were classified as NR. The overall response rate (ORR = CR + PR) and the CR rate in the thal-group were significantly higher than those in the ctrl-group (*p* < 0.05), whereas the NR rate was significantly lower in the thal-group (*p* < 0.05). Detailed efficacy outcomes are shown in Table 2.

**Table 2:** Responses and outcomes in the two groups.

Treatment response	ctrl-group (*n* = 47)	thal-group (*n* = 48)	*p*
OR	26 (55.32)	38 (79.17)	0.013
CR	7 (14.89)	21 (44.68)	
PR	19 (40.43)	17 (37.13)	
NR	21 (44.68)	10 (21.66)	

OR, overall response; CR, complete response; PR, partial response; NR, no response.

To further investigate the underlying factors contributing to the differences in treatment outcomes between the two groups, patients were stratified into CR, PR, and NR subgroups based on response to therapy. Comparative analyses were conducted for variables, including gender, age, pretreatment regimen, response to hormone therapy, bleeding score, baseline PLT count, and lymphocyte subpopulation profiles. No statistically significant differences were found in gender, age, pretreatment regimen, hormone response classification, baseline PLT count, or bleeding score among the CR, PR, and NR subgroups in either treatment group (*p* > 0.05). However, analysis of baseline lymphocyte subpopulations revealed that, in the thal-group, both the absolute count and percentage of CD16^+^CD56^+^CD3^−^ NK cells were significantly lower in the CR and PR subgroups compared to the NR subgroup (*p* < 0.05). No significant differences were found in total lymphocyte count, CD3^+^ T cells, CD4^+^ helper T cells, CD8^+^ cytotoxic T cells, or CD19^+^ B cells among these subgroups (*p* > 0.05). Similarly, in the ctrl-group, lower baseline levels of CD16^+^CD56^+^CD3^−^ NK cells were associated with improved response (CR + PR) compared to NR (*p* < 0.05). Detailed results are shown in Table 3.

**Table 3:** Lymphocyte subset data of ITP patients before and after rituximab and/or Thalidomide treatment.

Parameters	thal-group (*n* = 48)					ctrl-group (*n* = 47)				
	CR	PR	NR	t/χ^2^	*p*	CR	PR	NR	t/χ^2^	*p*
Number [*n* (%)]	21 (44.68)	17 (37.13)	10 (21.6)			7 (14.89)	19 (40.43)	21 (44.68)		
Gender (Male)	7 (33.33)	5 (29.41)	4 (40.00)	0.318^a^	0.853	2 (28.57)	7 (36.84)	11 (52.38)	1.643^a^	0.44
Age [M(P25, P75), years]	46.00 (33.75, 56.25)	45.00 (26.50, 55.00)	35.50 (22.25, 54.00)	1.745^b^	0.418	47.00 (29.00, 69.00)	53.00 (37.00, 59.00)	44.00 (29.50, 56.00)	0.193^b^	0.386
Treatment regimen before enrollment [*n* (%)]										
Glucocorticoids	21 (100.00)	17 (100.00)	10 (100.00)	-	-	7 (100.00)	19 (100.00)	21 (100.00)	-	-
Human immune disease globulin	2 (9.52)	3 (23.53)	3 (30.00)	2.063	0.356	1 (14.28)	2 (10.53)	2 (9.52)	0.126^a^	0.939
rhTPO	10 (47.62)	12 (70.59)	7 (70.00)	2.558^a^	0.278	5 (71.43)	14 (73.68)	16 (76.19)	0.073^a^	0.964
Hormonal response [*n* (%)]				0.318^a^	0.853				0.003^a^	0.999
Steroid-dependent	7 (33.33)	5 (29.41)	4 (40.00)			3 (42.86)	8 (42.11)	9 (47.37)		
Steroid-resistant	14 (66.67)	12 (70.59)	6 (60.00)			4 (57.14)	11 (57.89)	12 (63.16)		
Platelet count [M(P25, P75), 109/L]	17.50 (14.75, 21.0)	19.0 (17.00, 22.50)	18.50 (15.75, 23.25)	1.444	0.486	18.00 (14.00, 22.00)	19.00 (14.00, 22.00)	17.00 (16.00, 21.00)	1.354	0.618
Bleeding score [*n* (%)]				8.152^b^	0.419				8.933^b^	0.177
0	5 (23.81)	1 (5.88)	3 (30.00)			0	4 (21.05)	9 (42.86)		
1	10 (47.62)	9 (52.94)	2 (20.00)			4 (57.14)	7 (36.84)	9 (42.86)		
2	4 (19.05)	3 (117.65)	4 (40.00)			3 (42.86)	6 (31.58)	2 (9.52)		
3	2 (9.52)	3 (17.65)	1 (10.00)			0	2 (10.53)	1 (4.76)		
4	0	1 (5.88)	0			0	0	0		
Absolute value of lymphocyte subpopulations before treatment (cells/ul) Mean±SD										
Circulating lymphocytes	1682.86 ± 310.96	1708.94 ± 265.32	1833.90 ± 524.77	0.281	0.869	1839.25 ± 428.68	1642.74 ± 233.33	1688.95 ± 241.74	2.204	0.332
CD19 + B	368.00 ± 113.54	367.06 ± 140.36	206.53 ± 65.31	0.036	0.982	392.83 ± 200.88	336.88 ± 121.37	356.75 ± 135.56	0.51	0.775
Percentage of lymphocytes (%)	22.20 ± 7.35	21.72 ± 8.02	8.63 ± 2.73	0.028	0.986	20.69 ± 6.48	20.33 ± 6.21	21.51 ± 8.96	0.114	0.944
CD3 + T	1196.52 ± 300.01	1218.18 ± 280.27	1241.60 ± 370.26	0.015	0.992	1336.33 ± 281.60	1150.63 ± 202.84	1201.87 ± 361.24	3.932	0.14
Percentage of lymphocytes (%)	70.41 ± 7.72	70.77 ± 8.42	67.69 ± 7.69	1.261	0.535	73.25 ± 6.55	69.79 ± 4.66	69.98 ± 13.52	2.807	0.246
CD4 + helper T cells	631.95 ± 191.79	646.29 ± 146.55	668.90 ± 289.23	0.098	0.952	658.50 ± 282.78	526.44 ± 297.65	595.00 ± 267.39	1.53	0.465
Percentage of lymphocytes (%)	37.89 ± 11.06	38.49 ± 11.29	36.18 ± 10.50	0.234	0.89	38.53 ± 20.06	32.35 ± 18.66	35.17 ± 15.75	1.01	0.577
CD8 + cytotoxic T cells	592.14 ± 226.60	615.41 ± 266.06	601.70 ± 261.48	0.282	0.869	738.16 ± 367.03	649.85 ± 267.54	603.37 ± 330.68	0.536	0.765
Percentage of lymphocytes (%)	34.87 ± 10.20	35.62 ± 15.01	34.87 ± 10.20	0.093	0.955	39.98 ± 18.55	40.03 ± 15.65	34.62 ± 18.05	0.451	0.465
CD16 + CD56 + CD3- NK cells	78.81 ± 20.15	91.82 ± 28.64	139.00 ± 42.42^cd^	16.537^b^	0.001	81.00 ± 17.93	91.37 ± 26.54	163.62 ± 41.53^cd^	17.56^b^	0.001
Percentage of lymphocytes (%)	4.82 ± 1.57	5.55 ± 2.26	7.65 ± 1.41^c^	12.184^b^	0.002	4.83 ± 1.45	5.58 ± 1.81	10.05 ± 4.32^c^	12.32^b^	0.002

^a^ represents the *χ*^2^ value, ^b^ represents the H value, and the rest represent the *F* value. ^c^ Compared with the CR group, the corrected *p*-value was ≤0.0176 (after Bonferroni correction), ^d^ Compared with the PR group, *p* ≤ 0.0176 (after Bonferroni correction) indicates statistical significance. The remaining *p* < 0.05 also indicates statistical significance.

Following 6 months of treatment, post-treatment lymphocyte subset analysis showed no statistically significant differences in the percentage or absolute count of CD16^+^CD56^+^CD3^−^ NK cells among the CR, PR, and NR subgroups in the thal-group (*p* > 0.05). In the ctrl-group, a nonsignificant trend toward lower NK cell counts was observed in the NR subgroup compared to the CR and PR subgroups (*p* > 0.05). Full post-treatment lymphocyte data are provided in Table 4.

**Table 4:** Lymphocyte subset data of ITP patients at 6 months after rituximab and/or thalidomide treatment.

Absolute count of lymphocyte subpopulations after treatment (cells/ul) Mean±SD	thal-group (*n* = 48)					ctrl-group (*n* = 47)				
	CR (*n* = 21)	PR (*n* = 17)	NR (*n* = 10)	*F* value	*p*	CR (*n* = 7)	PR (*n* = 19)	NR (*n* = 21)	*F* value	*p*
Circulating lymphocyte count	1575.95 ± 271.14	1616.94 ± 271.21	1516.00 ± 220.62	1.372	0.503	1560.00 ± 259.26	1550.36 ± 203.72	1576.28 ± 195.75	0.390	0.823
CD19 + B	138.47 ± 59.16	135.05 ± 51.75	148.80 ± 77.04	0.043	0.978	111.57 ± 49.47	148.00 ± 61.37	95.46 ± 43.79	2.790	0.247
Percentage of lymphocytes (%)	8.89 ± 3.97	8.56 ± 3.41	9.66 ± 4.35	0.279	0.869	6.76 ± 3.06	8.58 ± 3.23	6.31 ± 3.64	4.514	0.104
CD3 + T	1205.80 ± 228.08	1236.11 ± 251.70	1154.60 ± 186.61	1.191	0.551	1232.57 ± 216.33	1169.52 ± 153.69	1214.38 ± 132.96	1.010	0.603
Percentage of lymphocytes (%)	76.65 ± 8.63	76.58 ± 8.94	76.40 ± 7.81	0.034	0.982	79.43 ± 8.79	76.05 ± 9.38	77.58 ± 7.64	1.778	0.410
CD4 + helper T cells	597.19 ± 184.16	626.88 ± 122.77	525.90 ± 191.93	2.587	0.274	590.71 ± 79.82	511.89 ± 99.39	537.19 ± 90.92	3.567	0.168
Percentage of lymphocytes (%)	37.72 ± 10.44	39.33 ± 7.86	34.56 ± 11.03	1.817	0.402	41.70 ± 7.63	33.39 ± 8.87	36.74 ± 7.37	5.415	0.067
CD8 + cytotoxic T cells	536.00 ± 168.41	581.00 ± 207.78	590.60 ± 213.28	0.593	0.743	587.42 ± 235.10	607.47 ± 187.93	629.42 ± 161.86	0.346	0.840
Percentage of lymphocytes (%)	34.61 ± 10.63	35.49 ± 10.29	39.25 ± 12.67	1.503	0.471	36.73 ± 10.55	39.05 ± 10.53	36.73 ± 9.36	0.406	0.815
CD16 + CD56 + CD3- NK cells	137.23 ± 17.42	144.52 ± 18.81	134.50 ± 18.36	2.223	0.328	131.57 ± 33.35	124.70 ± 25.79	126.80 ± 30.44	0.658	0.719
Percentage of lymphocytes (%)	8.95 ± 2.03	9.43 ± 2.14	9.13 ± 2.25	0.564	0.754	7.38 ± 4.17	6.32 ± 4.40	7.24 ± 5.06	1.311	0.519

**Figure 1:**
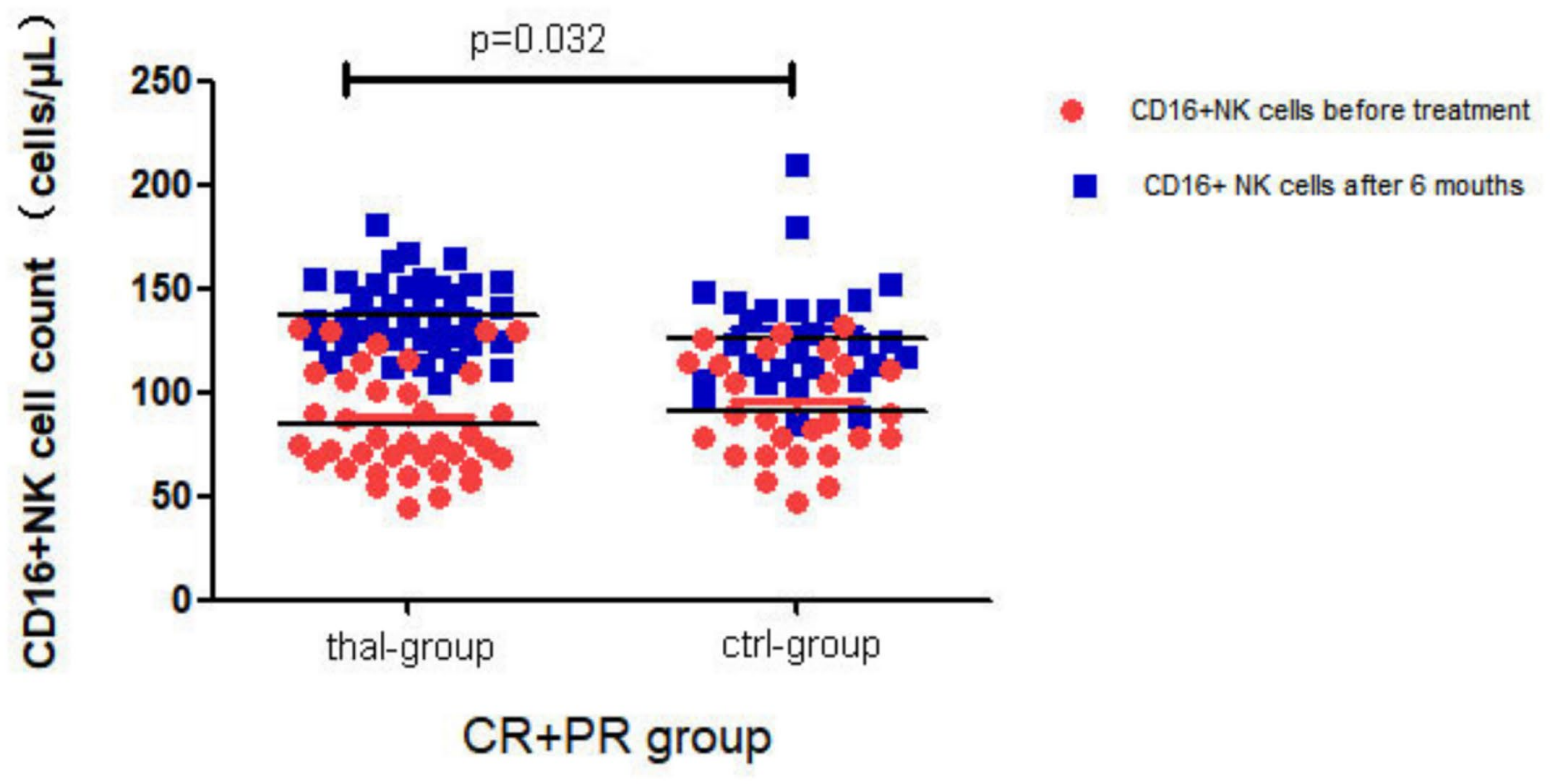
Number of circulating NK cells by the RTX response and/or thalidomide treatment.

To further elucidate immunological changes associated with treatment response, patients in both the thal-group and ctrl-group were stratified into overall response (OR: CR + PR) and NR subgroups. Longitudinal changes in lymphocyte subsets, including CD19^+^ B cells, CD3^+^ T cells, CD3^+^CD4^+^ helper T cells, CD3^+^CD8^+^ cytotoxic T cells, and CD16^+^CD56^+^CD3^−^ NK cells, were analyzed before and after treatment. Among patients in the thal-group who achieved OR, the mean ± standard deviation (SD) of CD16^+^ NK cell count increased significantly from 84.63 ± 24.85 cells/μL before treatment to 138.71 ± 19.20 cells/μL after 6 months (*z* = −5.272, *p* < 0.001). In contrast, no statistically significant change was observed in the NR subgroup of the same treatment group (*z* = −0.392, *p* = 0.695). Similarly, in the ctrl-group, patients in the OR subgroup exhibited a significant increase in NK cell count from 92.96 ± 23.17 cells/μL to 129.73 ± 28.50 cells/μL after treatment (z = −3.889, *p* < 0.001), while no significant change was observed in the NR subgroup (z = −1.147, *p* = 0.251). Comparative analysis between treatment groups revealed no significant difference in baseline NK cell counts in the OR subgroup (*t* = −1.353, *p* = 0.181). However, after 6 months, the NK cell count in the thal-group was significantly higher than in the ctrl-group (*t* = 2.036, *p* = 0.032). No statistically significant differences were observed in the counts of CD19^+^ B cells, CD3^+^ T cells, CD3^+^CD4^+^ T cells, or CD3^+^CD8^+^ T cells before and after treatment in either group among OR patients (*p* > 0.05). These results are illustrated in Figure 1.

### 3.3 Comparison of ROC curves for patients

**Figure 2:**
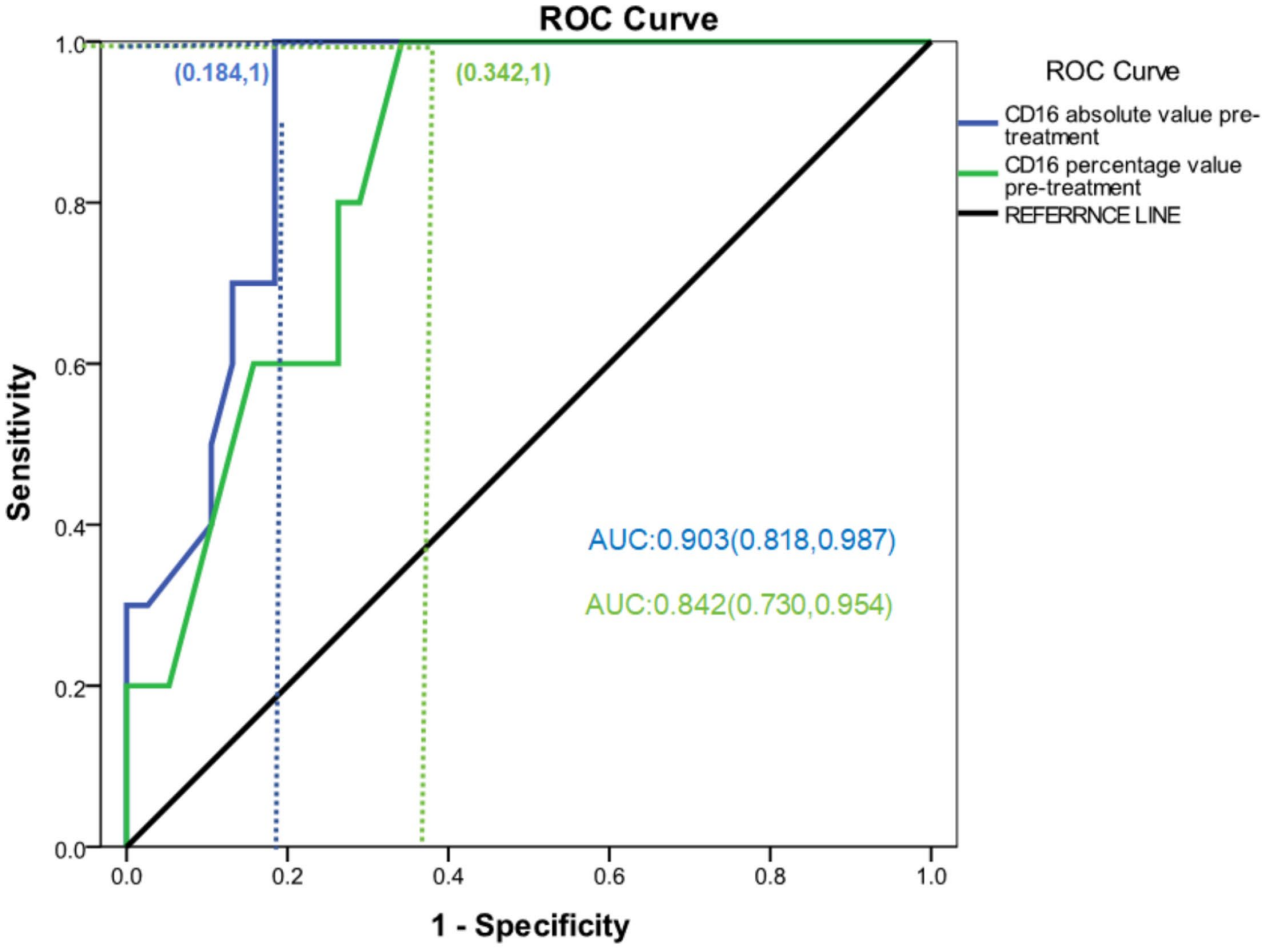
ROC curves of absolute value and percentage of NK cells were plotted in CR + PR group and NR group.

To evaluate the predictive value of CD16^+^CD56^+^CD3^−^ NK cell levels for treatment response, ROC curves were generated to distinguish responders (CR + PR) from NR in both the thal-group and ctrl-group, based on the absolute count and percentage of NK cells before treatment. As described in Section 2.2, significant differences in NK cell levels were observed between responders and nonresponders across both treatment arms. The area under the ROC curve (AUC) for the absolute NK cell count before treatment was 0.903 (*p* < 0.01), and for the NK cell percentage, the AUC was 0.846 (*p* < 0.01), both significantly different from the reference value of AUC = 0.5, indicating a discriminatory capacity for predicting treatment response. Optimal cutoff values were determined using the Youden index, which balances sensitivity and specificity. When the absolute NK cell count threshold was set at 110.5 cells/μL, the sensitivity was 81.6%, the specificity was 100.0%, and the Youden index was 0.816. For the percentage of NK cells, a cutoff value of 5.72% yielded a sensitivity of 65.8%, specificity of 100.0%, and a Youden index of 0.658. These findings suggest that both the absolute and relative levels of CD16^+^CD56^+^CD3^−^ NK cells prior to treatment may serve as useful immunological biomarkers for predicting response to LD-RTX alone or in combination with THD in steroid-resistant or steroid-refractory ITP (Figure 2).

### 3.4 Adverse reactions (AEs)

AEs were primarily of mild to moderate severity (grades 1–2) in both groups. In the thal-group, the most common AEs were respiratory tract infections, reported in 17 out of 48 patients (35.42%), and somnolence, reported in 8 out of 48 patients (16.67%). In the ctrl-group, respiratory tract infections were the only notable AE, observed in 13 out of 47 patients (27.66%). One patient in the combination group developed a pulmonary infection after receiving three doses of RTX and was unable to continue scheduled treatment, resulting in withdrawal from the study. Another patient in this group experienced hemoptysis in the eighth week of enrollment and similarly discontinued trial participation, opting for alternative therapy. In the ctrl-group, two patients developed pulmonary infections after the first and second RTX infusions, respectively, leading to early discontinuation. Additionally, one patient withdrew due to severe gastrointestinal bleeding, which was attributed to persistently low PLT counts, possibly indicating treatment resistance. Overall, the incidence of AEs in the two groups is not significant. A detailed summary of adverse reactions is provided in Table 5.

**Table 5:** Adverse events recorded in the two groups.

Parameters	thal-group (*n* = 48)	ctrl-group (*n* = 47)
Death (%)	0	0
Adverse reactions *n* (%)	20 (41.67)	16 (34.04)
Peripheral neuropathy *n* (%)	5 (10.42)	0
Respiratory infection *n* (%)	17 (35.42)	13 (27.66)
I	10 (20.83)	7 (14.89)
II	5 (10.42)	4 (8.51)
III	2 (4.17)	3 (6.38)
Constipation *n* (%)	4 (8.33)	0
Dry skin and mucous membrane (*n*%)	1 (2.08)	0
Somnolence *n* (%)	8 (16.67)	0
Fever *n* (%)	4 (8.33)	3 (6.38)
Grade 3–4 adverse events (AEs)		
Pneumonia *n* (%)	1 (2.08)	2 (4.26)
Bleeding (%)	1 (2.08)	1 (2.13)

## 4 Discussion

This study enrolled a total of 100 patients who were randomly assigned to receive either LD-RTX alone or T + LD-RTX. At the 6-month follow-up, the ORR in the ctrl-group was 55.32%, which is consistent with previous domestic and international studies, reaffirming that the therapeutic efficacy of ctrl-group remains limited. In contrast, the group receiving THD in combination with LD-RTX demonstrated a significantly higher ORR of 79.17% (*p* < 0.05), indicating a clear clinical benefit of the combination strategy. Previous studies have shown that THD can upregulate the expression of NRP-1 and increase the number of Tregs in ITP patients, and induce response in corticosteroid-resistant or recurrent ITP patients (8). Ma J et al. (9) demonstrated that THD can correct the decreased proliferation ability of MSCs in ITP patients and enhance immune suppression.

In terms of safety, there was no significant difference in the overall incidence of adverse reactions between the thal-group (41.67%) and the ctrl-group (34.04%), indicating that the combination of thalidomide did not lead to a sharp increase in adverse reactions. However, there are significant differences in the characteristic spectra of adverse reactions between the two groups. This difference was mainly reflected in specific toxicities related to thalidomide: peripheral neuropathy, somnolence, and constipation were observed only in the combination therapy group, while no such events were reported in the control group receiving LD-RTX alone. The most common adverse reactions in the thal-group were drowsiness and respiratory infections, while the ctrl-group mainly experienced respiratory infections. Peripheral neuropathy (10.42%) is one of the most common and concerning dose-limiting toxicities of thalidomide, with an incidence rate of 10.42% consistent with literature reports (18). Sleepiness (16.67%) may be due to the clear central sedative effect of thalidomide (19). Constipation (8.33%) and dry skin and mucous membranes (2.08%) are anticholinergic side effects of thalidomide (20). The incidence of respiratory infections in the thal-group (35.42%) was slightly higher than that in the ctrl-group (27.66%), which may be related to mild immune suppression caused by the immunomodulatory effect of thalidomide, increasing susceptibility to common community-acquired infections (21). The incidence of pneumonia in the ctrl-group (6.38%) is actually higher than that in the thal-group (2.08%), which may be related to the small sample size, and individual events can lead to significant fluctuations in the rate. The most commonly observed adverse reactions in the combination group were somnolence and respiratory tract infection, whereas the ctrl-group mainly experienced respiratory tract infections. A majority of AEs were mild to moderate (grades 1–2), and the combination regimen was generally well tolerated. These findings indicate that T + LD-RTX significantly improves therapeutic efficacy while maintaining a safety profile that is predictable and manageable. The treatment-emergent toxicities primarily consisted of established side effects of thalidomide and did not lead to severe safety risks, supporting this regimen as a more promising strategy for treating refractory or steroid-resistant ITP.

To further investigate the potential mechanism underlying the enhanced efficacy of combination therapy, we conducted a preliminary analysis of peripheral lymphocyte subsets in 100 patients with steroid-dependent or refractory ITP before treatment. Baseline comparisons showed no significant differences between the ctrl-group and thal-group in the absolute count or percentage of lymphocyte subsets, including CD16^+^CD56^+^CD3^−^ NK cells. However, subgroup analysis revealed that patients who achieved a CR or PR had significantly lower pre-treatment levels of CD16^+^CD56^+^CD3^−^ NK cells compared with NR. This finding suggests that lower baseline NK cell levels may serve as a predictive biomarker of treatment responsiveness to LD-RTX or T + LD-RTX therapy. Furthermore, longitudinal follow-up demonstrated a significant post-treatment increase in NK cell counts in both treatment groups, with a more pronounced elevation observed in the thal-group. These results imply a potential association between therapeutic efficacy and NK cell recovery, particularly in the combination therapy group. We hypothesize that NK cells may exert a protective effect in ITP by attenuating PLT destruction, thereby contributing to hematologic remission. This mechanistic insight aligns with the findings of Etienne Rivière et al. (22), who reported a role for NK cells in modulating immune-mediated thrombocytopenia.

The pathogenesis of ITP involves a complex interplay of immune dysregulation, in which NK cells play an increasingly recognized role (23). NK cells contribute to immune homeostasis by limiting the presentation of self-antigens and regulating the adaptive immune response, primarily through the elimination of immature dendritic cells that present self-antigens and the secretion of immunoregulatory cytokines (24). Zhang Yujiao et al. (25) reported that patients with ITP exhibit reduced NK cell counts, impaired maturation, and diminished cytotoxic function, which may result in insufficient suppression of autoreactive T and B lymphocytes, thereby promoting disease progression. Further evidence from RM Talaat et al. (26), who studied pediatric acute and chronic ITP, showed that both Treg and NK cells were significantly reduced compared to healthy controls. These findings suggest that loss of immune tolerance, including dysfunction or depletion of NK cells, may play a central role in ITP pathophysiology. In particular, chronic overactivation of the immune system in steroid-dependent ITP may lead to NK cell exhaustion, ultimately disrupting immune regulation (27). This dysregulation may result in a cascade of adaptive immune dysfunction, including T/B-cell hyperactivity, cytokine imbalance, and impaired immune cell proliferation and activation. THD has been reported to modulate the cytokine environment, particularly by regulating the secretion of TNF-*α*, IL-2, and IL-12, and thereby reshape the immune microenvironment and promote NK cell proliferation and activation (28, 29). Additional studies have suggested that THD may exert direct effects on NK cell precursors in the bone marrow, facilitating their differentiation and increasing peripheral NK cell counts (7, 25). Furthermore, PTX (LD-RTX) has been shown to enhance NK cell-mediated cytotoxicity, particularly in the presence of T-cell activation (30). In our study, the combination of THD and PTX (T + LD-RTX) considerably increased peripheral blood CD16^+^CD56^+^CD3^−^ NK cell counts in patients with steroid-refractory or steroid-dependent ITP, compared to monotherapy. This finding supports the hypothesis that the synergistic effects of THD and PTX on NK cell recovery may underlie the improved therapeutic outcomes observed in the combination therapy group.

The findings of this study demonstrate that, among patients who achieved an OR, both the absolute count and percentage of CD16^+^CD56^+^CD3^−^ NK cells differed significantly between the thal-group and ctrl-group when compared to NR. Notably, patients who achieved CR or PR exhibited lower baseline levels of NK cells, suggesting that, in patients with steroid-resistant or refractory ITP, therapeutic efficacy may be inversely correlated with pretreatment NK cell levels. ROC curve analysis further supported this relationship, indicating that lower NK cell percentages were predictive of CR/PR, thus identifying a potential biomarker for treatment response.

However, the present study has several limitations. The sample size was relatively small, which may affect the statistical power and generalizability of the results. The sample size of this study is not based on pre-calculated statistical efficacy, so caution should be exercised when interpreting comparisons between treatment groups. The results are mainly used to generate hypotheses rather than draw conclusive conclusions. This study lacks long-term follow-up data for patients after 6 months, and due to unclear long-term efficacy and safety, it is difficult for us to make a comprehensive judgment on the ultimate value of this combination therapy in clinical practice. In addition, the design of this study focuses on evaluating the effectiveness and overall safety of low-dose rituximab combined with thalidomide in real-world clinical practice. Routine clinical follow-up data do not systematically include deep immune monitoring indicators such as lymphocyte subset counts; therefore, we cannot accurately describe the broader immune suppression state induced by this combination regimen. Despite this limitation, the clinical efficacy data provided in this study provide preliminary evidence for further exploration of this combination regimen. Future research should conduct larger-scale, multi-center, multi-population, cross-regional, prospective studies. In addition, extended follow-up periods are essential to evaluate the long-term efficacy, durability of response, and safety of T + LD-RTX combination therapy. These efforts will be critical in optimizing individualized treatment strategies and improving clinical outcomes for patients with ITP.

## 5 Conclusion

T + LD-RTX treatment considerably improved OR in patients with steroid-refractory or steroid-dependent ITP, with an acceptable and manageable safety profile. Notably, the lower baseline levels of peripheral blood CD16^+^CD56^+^CD3^−^ NK cells were associated with a higher likelihood of achieving clinical response, suggesting their potential utility as a predictive biomarker for treatment efficacy. This combination therapy may exert its therapeutic effect by increasing circulating NK cell counts, contributing immune regulation, and preserving PLT count. Given its efficacy, safety, and cost-effectiveness, THD + LD-RTX treatment represents a promising treatment strategy for patients with difficult-to-treat ITP.

## Data Availability

The raw data supporting the conclusions of this article will be made available by the authors, without undue reservation.
